# mSphere of Influence: How host genetics impact microbial pathogenesis and treatment of infectious disease

**DOI:** 10.1128/msphere.00629-23

**Published:** 2023-12-14

**Authors:** Emily E. Rosowski

**Affiliations:** 1 Department of Biological Sciences, Clemson University, Clemson, South Carolina, USA; 2 Eukaryotic Pathogens Innovation Center, Clemson University, Clemson, South Carolina, USA; University of Georgia, Athens, Georgia, USA

**Keywords:** host-pathogen interactions, *Mycobacterium*, *Aspergillus*, zebrafish

## Abstract

Emily Rosowski works in the field of host-pathogen interactions, studying how host innate immune mechanisms control pathogens. In this mSphere of Influence article, she reflects on how “Host genotype-specific therapies can optimize the inflammatory response to mycobacterial infections” by D. M. Tobin, F. J. Roca, S. F. Oh, R. McFarland, et al. (Cell 148:434–446, 2012, https://doi.org/10.1016/j.cell.2011.12.023) made an impact on her by investigating how differences in host genetics can affect modes of microbial pathogenesis and inform treatments for infectious disease.

## COMMENTARY

In “Host genotype-specific therapies can optimize the inflammatory response to mycobacterial infections,” Tobin et al. aimed to identify and determine the mechanism of host genetic factors that drive susceptibility to mycobacterial infections (1). Mycobacterial infections, including tuberculosis, are often termed “opportunistic infections,” meaning that these microbes only have pathogenic potential in a subset of patients (2, 3). While these microbes generally affect immunosuppressed individuals, uncontrolled inflammation can also cause susceptibility. Hosts experience disease due to multiple underlying causes whereby host factors cause a range of immune responses that promote disparate modes of microbial pathogenesis. The key conclusion from this paper is that treatment efficacy can be dictated by host genetics. Most current therapies for infectious diseases focus on targeting the microbe, but this study demonstrates the importance of optimizing the host response to infection.

To identify host genetic factors that drive susceptibility to mycobacterial infections, Tobin et al. used a larval zebrafish*-Mycobacterium marinum* infection system, which recapitulates many aspects of human tuberculosis. They had previously used a forward genetic screen to identify susceptible mutants, finding mutations in *lta4h* which encodes an enzyme that synthesizes a pro-inflammatory leukotriene, LTB4 (4). The authors determined that when Lta4h isn’t functional, precursor lipids get diverted into a lipoxin pathway, generating an excess of the anti-inflammatory lipoxin LXA4 (4). In this follow-up paper, Tobin et al. delved into how alterations to leukotriene and lipoxin levels drive susceptibility to *M. marinum* (1). Using the genetic tools available in zebrafish, they generated both “Lta4h low” and “Lta4h high” hosts. Importantly, both of these genetic alterations lead to increased susceptibility to *M. marinum* and increased bacterial growth, but the modes of pathogenesis are different. In the “Lta4h low” condition, Tnf levels are low, and macrophages fail to control intracellular bacteria that grow and lyse out of these cells. In the “Lta4h high” condition, Tnf levels are high, and macrophages can control bacterial growth early in infection, but these cells later die through necrosis, releasing bacteria.

Tobin et al. then ask how these two genetically different, but equally susceptible, hosts respond to treatment. In human patients with meningeal tuberculosis, the corticosteroid dexamethasone is used to control inflammation in the brain. When infected larval zebrafish were treated with dexamethasone, it controlled bacterial load in “Lta4h high” animals but made the infection worse in “Lta4h low” animals. The authors identified an SNP in the promoter of the human *LTA4H* gene that affects gene expression. In a cohort of patients from Vietnam with meningeal tuberculosis, they retrospectively analyzed patient survival based on SNP genotype. Overall, they found a heterozygote advantage, in line with the idea that both too little and too much inflammation can be detrimental. The most striking results were seen when they focused on patients who were treated with dexamethasone. All patients homozygous for the *LTA4H* high-expression SNP survived after dexamethasone treatment. However, in patients homozygous for the *LTA4H* low-expression SNP, dexamethasone decreased overall survival, demonstrating that genetic backgrounds can be differentially responsive to the same treatment.

I have been interested in host-pathogen interactions since my PhD training, where I studied how strains of *Toxoplasma gondii* differentially activate host pathways, including NF-κB (5). *T. gondii* can infect a huge range of hosts, and I was struck by the idea that genetically diverse strains of *T. gondii* have evolved to survive and grow in hosts with different immune responses (6). I was interested in studying these specific host-pathogen interactions—how differences in host immune responses allow microbes to grow in specific contexts—but my training thus far had focused on understanding the pathogen side of this equation. This study by Tobin et al. strongly influenced my decision to learn the larval zebrafish model to study the host side. In addition to demonstrating the application of zebrafish research to human disease, this paper exemplifies three of the key strengths of the zebrafish model for host-pathogen research: (i) the array of genetic resources available, (ii) live imaging of larvae to characterize infection progression, and (iii) the simple application of drugs to determine treatment efficacy.

In my laboratory, we use larval zebrafish to study innate immune responses to *Aspergillus* fungi. Like mycobacteria, *Aspergillus* infections impact patients with altered immune responses, and we are interested in understanding the cellular and molecular causes of susceptibility in different host backgrounds. For example, individuals with chronic granulomatous disease (CGD) are susceptible to *Aspergillus nidulans*, a species of *Aspergillus* that does not affect patients with other risk factors, and we found that the fast germination rate of *A. nidulans* leads to clearance in wild-type hosts but drives excessive inflammation in CGD zebrafish (7). I am also interested in understanding the direct causes of susceptibility in patients treated with immunosuppressive drugs. The anti-inflammatory drug indomethacin decreases the survival of larvae infected with *Aspergillus fumigatus*, but we found that this drug has no significant impact on the ability of macrophages or neutrophils to respond to the infection or kill fungal spores (8). Instead, indomethacin inhibits immune control of later infection stages, including spore germination and invasive hyphal growth (8). We are also investigating how different host susceptibility factors impact the efficacy of anti-fungal therapies. We found that, in larval zebrafish, voriconazole does not inhibit the germination of *A. fumigatus* spores but instead collaborates with macrophages to destroy fungal hyphae (9), and we are now expanding these studies to more immunosuppressed host backgrounds and anti-fungal drugs.

The idea of targeting the host to improve survival to pathogenic microbes is becoming more accepted (10, 11); however, these approaches rely on understanding the specific host-pathogen context of each patient. This paper by Tobin et al. demonstrates the utility of the larval zebrafish in determining how different host genetic backgrounds impact the mode of microbial pathogenesis and treatment efficacy, an approach that we are currently using in my laboratory.
